# A Sentinel-2 benchmark dataset for deep-learning active-fire segmentation across 25 California wildfires, July 2020–August 2026

**DOI:** 10.1016/j.dib.2026.113357

**Published:** 2026-10-15

**Authors:** Shreyan Mitra, Mohammadreza Narimani, Parastoo Farajpoor

**Affiliations:** a California High School, San Ramon, CA, 94583, USA; b Department of Biological and Agricultural Engineering, University of California, Davis, CA, 95616, USA

**Keywords:** Remote sensing, Weak supervision, Shortwave infrared, Image masks, Rare-class labeling, Grouped evaluation, Algorithmic annotation

## Abstract

This article describes an open image dataset for active-fire segmentation in satellite imagery. It contains 2148 image-mask pairs from 25 California wildfires, acquired from 23 July 2020 to 21 August 2026. Each image is a 512 × 512-pixel, three-channel composite of Sentinel-2 Level-2A bands B12, B11 and B8A at 20 m sampling. The same linear rendering is applied to every image. Masks distinguish background, SWIR-rule active fire and invalid observations. They were generated from shortwave-infrared brightness and near-infrared contrast, followed by two steps of neighborhood growth. The release includes chip-level metadata and a partition with 18 training, three validation and four test wildfires. Each wildfire belongs to only one partition. Of the image pairs, 841 contain active-fire labels, which occupy 0.0766% of all grid cells. An analyst reviewed 233 test chips without seeing the rule masks. These annotations provide a separate assessment of label agreement at chip and connected-component levels. The accompanying code includes a ResNet-34 U-Net with checkpoint and probability-threshold selection on validation data. The images and masks provide a fixed input for comparing segmentation methods on wildfires excluded from training. Dataset version 1.0.0 is deposited on Zenodo; the preparation and training software is available on GitHub.

Specifications Table

**Table:** 

Subject	Earth and Environmental Sciences
Specific subject area	Satellite remote sensing; active-fire segmentation; deep learning with algorithmic supervision
Type of data	Processed 8-bit RGB PNG images; single-channel integer PNG masks; CSV metadata; analyst annotations; descriptive figures; Python software. Source reflectance assets were accessed during construction and are not part of the distributed image representation.
Data collection	Sentinel-2 Level-2A (Earth Search, sentinel-2-l2a) and NIFC WFIGS were used. We retained 25 California wildfires discovered after 1 January 2019 with area >20,000 acres. Scenes intersected each fire’s bounding-box midpoint; cloud cover was ≤95%. Up to 14 dates were sampled from discovery to containment (60-day cap; 45-day fallback; 7-day minimum). At 20 m, 1536 × 1536-cell windows were cut into nine 512 × 512 chips. Chips >50% invalid were removed; all fire-bearing chips and subsampled fire-free chips were kept. B12/B11/B8A formed the images; Eqs. (2)–(4) defined the masks. Full criteria are in Methods.
Data source location	California, United States (Fig. 2); preparation and analysis at University of California, Davis, Davis, CA 95616, USA. Acquisitions span 23 July 2020 to 21 August 2026.
Data accessibility	Repository name: Zenodo Data identification number: 10.5281/zenodo.22713948 [1] Direct URL to data: https://doi.org/10.5281/zenodo.22713948 Instructions for accessing these data: Public access; data are licensed CC BY 4.0. Companion software is available from GitHub [2] under the MIT license (commit e5e466787ee7cae21268224b33a23df2901df406).
Related research article	None.

## 1 Value of the Data

• The release contains 2148 paired PNG files: 512 × 512 images in images/ and masks with matching basenames in masks/. The summary.csv file records the wildfire name, acquisition date, days since discovery, fire-pixel count and percentage of invalid cells for each chip. These files let researchers load the same image-mask pairs and examine class imbalance without downloading and processing the source Sentinel-2 scenes again.
• The partitions.csv file assigns each wildfire to training, validation or test. The sets contain 18, three and four wildfires, respectively, with 1470, 246 and 432 chips. Castle, McCash and Bobcat are reserved for validation; Slater, Hopkins, CALDWELL and Windy are reserved for testing. All chips from a wildfire remain in the same set. Researchers can therefore compare methods on the same held-out wildfires using the published partition.
• Fire labels occur in 841 chips and occupy 431,427 grid cells, or 0.0766% of the archive. Masks encode background as 0, SWIR-rule active fire as 1 and invalid observations as 255. The archive includes 1307 fire-free chips. These counts and the documented label rule provide a reference for testing class weighting, sampling and decision thresholds when fire pixels are rare. The prevalence denominator includes all grid cells; training and reported evaluation exclude cells coded 255.
• The analyst_review/hand_masks/ directory contains annotations for 233 of the 432 test chips. The analyst inspected the images without seeing the generated masks. Researchers can use these annotations to assess agreement with the SWIR rule at chip and connected-component levels. The annotations remain separate from the original rule masks, and cells marked as uncertain are excluded from agreement calculations.
• The public repository [2] supplies build_fire_dataset.py for preparing the archive and kaggle_fire_train.py for reference training. Table 4 records the training settings, and docs/REFERENCE_RUN.md documents the reported run. The Zenodo deposit [1] fixes the data at version 1.0.0, while commit e5e466787ee7cae21268224b33a23df2901df406 identifies the code used for this article. Using these versions keeps method comparisons tied to the same files and processing rules; new queries to the source services may return different scenes.

## 2 Background

An active-fire mask identifies where a satellite observation contains evidence of ongoing combustion. It does not map the area eventually burned. Sentinel-2 provides shortwave-infrared observations at 20 m sampling for locating active fire [3]. To use these observations for segmentation, researchers need to know how scenes were selected, how labels were generated and how images were assigned to training and evaluation sets.

Existing resources address fire detection at different observation scales. Pereira et al. [4] developed a Landsat-8 active-fire dataset with algorithmic annotations and a separate manually annotated evaluation set. Fire-video annotations provide frame-level masks for camera-based surveillance [5]. Geospatial datasets record the timing, location and extent of wildfire and prescribed-fire activity [6]. We compiled Sentinel-2 image-mask pairs with fixed rendering, partitions by wildfire name and a separate analyst review. The dataset provides a reference for pixel classification with sparse, algorithmically generated labels. The accompanying model is a reuse example.

## 3 Data Description

### 3.1 Release organization and partition inventory

The dataset is archived as version 1.0.0 on Zenodo [1]. It contains paired image and mask directories, a chip manifest, a partition file and analyst-review masks. The public repository [2] provides the construction, training and annotation-import scripts. Table 1 lists the chips and fire pixels in each partition. Fig. 1 shows chip counts, wildfire counts and the proportion of grid cells labeled as fire.

**Table 1:** Partition inventory by wildfire name. Fire prevalence uses all 512 × 512 grid cells in the denominator, including cells encoded as invalid. Training and evaluation exclude invalid cells.

Partition	Wildfires	Chips	Fire-bearing chips	Fire pixels	Fire prevalence (%)
Training	18	1470	548	284,948	0.0739
Validation	3	246	137	75,028	0.1163
Test	4	432	156	71,451	0.0631
Total	25	2148	841	431,427	0.0766

**Fig. 1:**
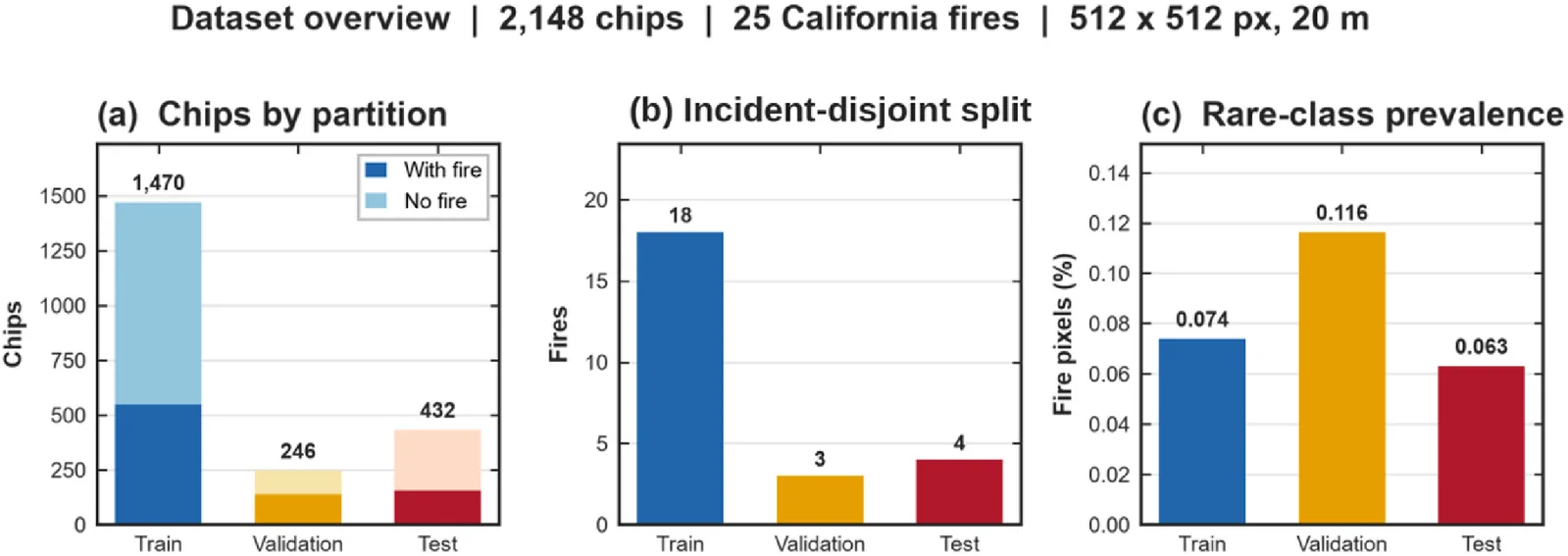
Dataset and partition inventory. (a) Chip counts, subdivided into chips with and without rule-generated fire labels. (b) Numbers of wildfires in the training, validation and test partitions. (c) Fire-pixel prevalence as a percentage of all grid cells in each partition. Dark and light portions in panel (a) denote fire-bearing and fire-free chips, respectively. Each wildfire is assigned to only one partition: training, validation or test. Chips from the same wildfire are never split across these sets.

All observations from Castle, McCash and Bobcat belong to validation. Slater, Hopkins, CALDWELL and Windy form the test partition; the remaining 18 wildfires form the training partition. No wildfire name occurs in more than one set. The archive contains 1307 fire-free chips and 841 fire-bearing chips. It is therefore not balanced at the chip level. These counts describe the selected samples and do not estimate how frequently fire occurs in California satellite observations.

Each image and its corresponding mask share a basename of the form {fire_slug}_{YYYY-MM-DD}_r{row}c{column}.png. The row and column indices range from 0 to 2 within a sampled parent window. A file can therefore be associated with its incident, observation date and position in that window without opening the image.

### 3.2 Spatial and temporal coverage

Fig. 2 locates the 25 wildfire perimeters and shows their partition assignments. Here, an incident means one named wildfire in the WFIGS records. Perimeters provide the spatial context for sampling, while the active-fire labels come from the SWIR rule. Each chip spans 10.24 km, and each parent window spans 30.72 km. The release contains repeated observations around the selected wildfires. It is not a continuous statewide mosaic.

**Fig. 2:**
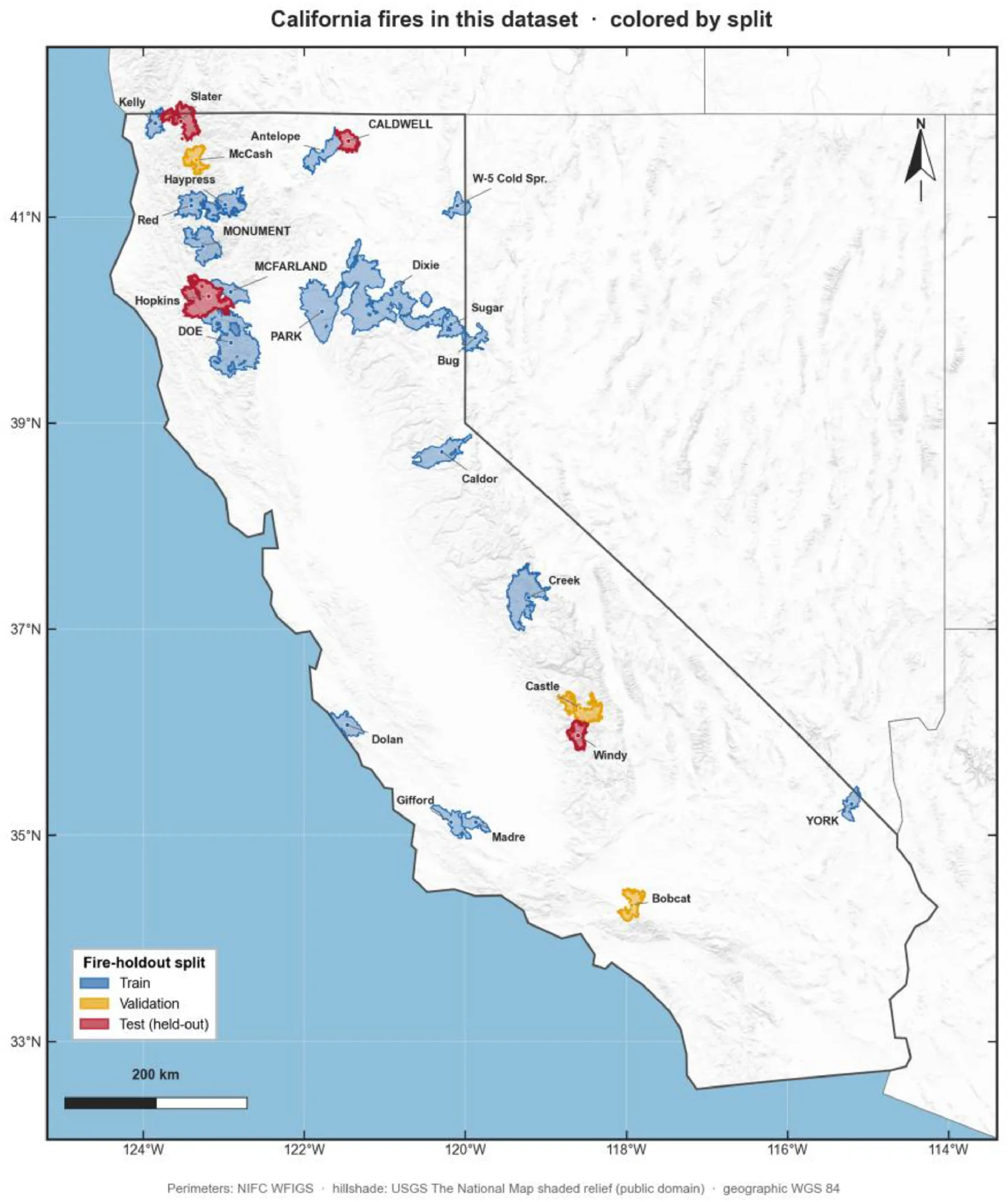
Locations of the 25 California wildfires in this dataset, colored by partition: training (blue), validation (yellow) and test (red). Perimeters are from NIFC WFIGS; the background is USGS The National Map shaded relief (public domain). Geographic coordinates provide spatial reference. Perimeters depict wildfire extents, not instantaneous flame fronts. Map lines delineate study areas and do not necessarily depict accepted national boundaries.

### 3.3 Image channels, label encoding and metadata

The RGB channels contain B12, B11 and B8A, respectively. B12 and B11 are shortwave-infrared bands, and B8A is the narrow near-infrared band. All three are sampled at their native 20 m spacing [3]. The same rendering limits are used for every chip. Table 2 describes the files and metadata needed to read the release.

**Table 2:** File and metadata dictionary. The summary.csv table has one row per distributed chip. Partition membership is supplied separately and is also derived from incident names by the training script.

File or field	Interpretation
images/*.png	512 × 512 × 3 arrays, unsigned 8-bit integers; channels B12, B11, B8A.
masks/*.png	512 × 512 arrays; 0 = background, 1 = SWIR-rule active fire, 255 = invalid observation.
summary.csv: file_name	Shared image-mask basename.
summary.csv: fire; date	Wildfire name; acquisition date in YYYY-MM-DD format.
summary.csv: day_of_burn	Integer days since the recorded discovery date; not a direct measure of local combustion duration.
summary.csv: fire_px; nodata_pct	Number of value-1 mask cells; percentage of cells encoded 255.
partitions.csv	Assignment of wildfire names to training, validation or test.
analyst_review/hand_masks/	Review masks for 233 test chips; 255 also represents analyst uncertainty.

The label rule uses shortwave-infrared contrast for hot-target detection [7]. Fig. 3 shows converted L2A band-value distributions for B8A, B11 and B12 in hand-drawn class regions from a Castle chip. Fig. 4 presents four image-mask examples. The true-color views provide visual context and are not part of the distributed three-channel images.

**Fig. 3:**
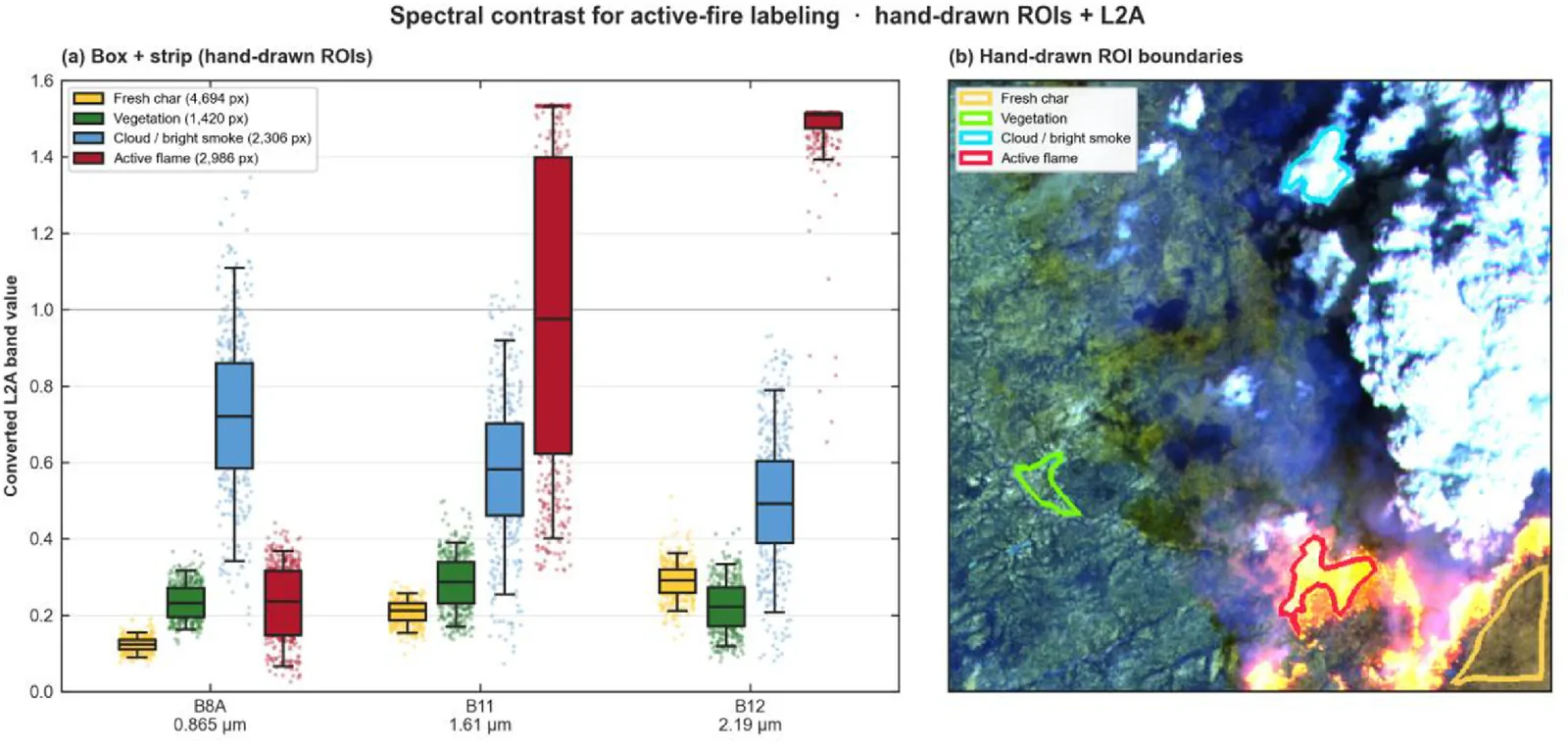
Spectral contrast associated with active-fire labeling. (a) Distributions of converted Sentinel-2 Level-2A B8A, B11, and B12 band values extracted from hand-drawn regions representing fresh char, vegetation, cloud/bright smoke, and active flame in a Castle-fire image. (b) Locations of the corresponding hand-drawn regions on the SWIR composite. Values represent discrete band measurements rather than continuous spectral signatures. The algorithmic fire-label rule is defined separately in Eqs. (2)–(4).

**Fig. 4:**
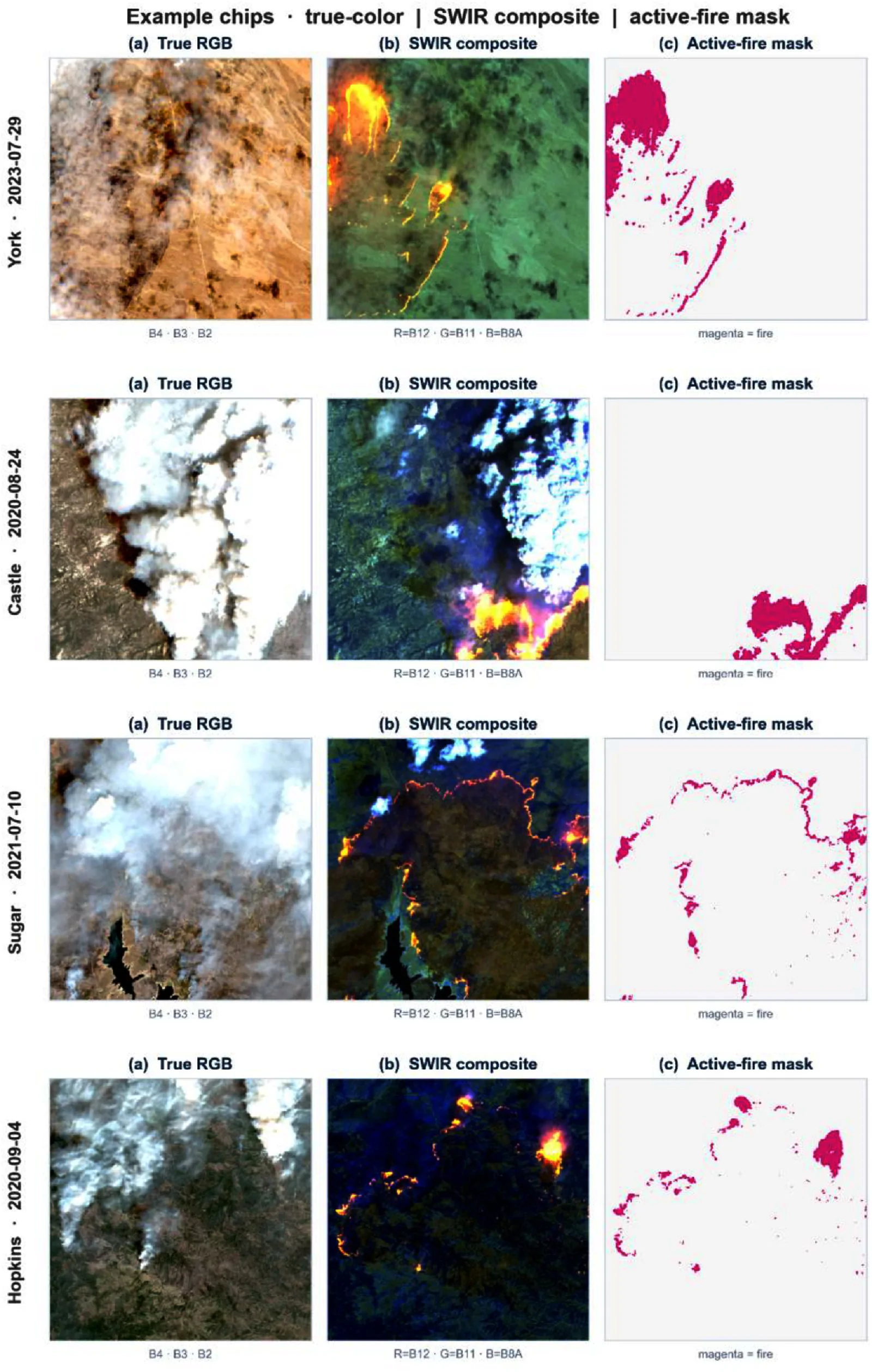
Examples from York (29 July 2023), Castle (24 August 2020), Sugar (10 July 2021) and Hopkins (4 September 2020). Columns show true color (B4-B3-B2), the SWIR composite (B12-B11-B8A) and the rule-generated fire mask in magenta. True-color panels are contextual illustrations, not additional channels in the released three-channel PNGs. Display colors do not change the integer encoding of the distributed masks.

### 3.4 Positive-class prevalence and spatial structure

Across the release, 431,427 grid cells carry active-fire labels. A total of 841 chips, or 39.2% of all chips, contain at least one such cell. The median fire-bearing chip contains 79 fire-labeled cells. Fig. 5a describes the distribution across positive chips, and Fig. 5b describes connected-component sizes in the test partition.

**Fig. 5:**
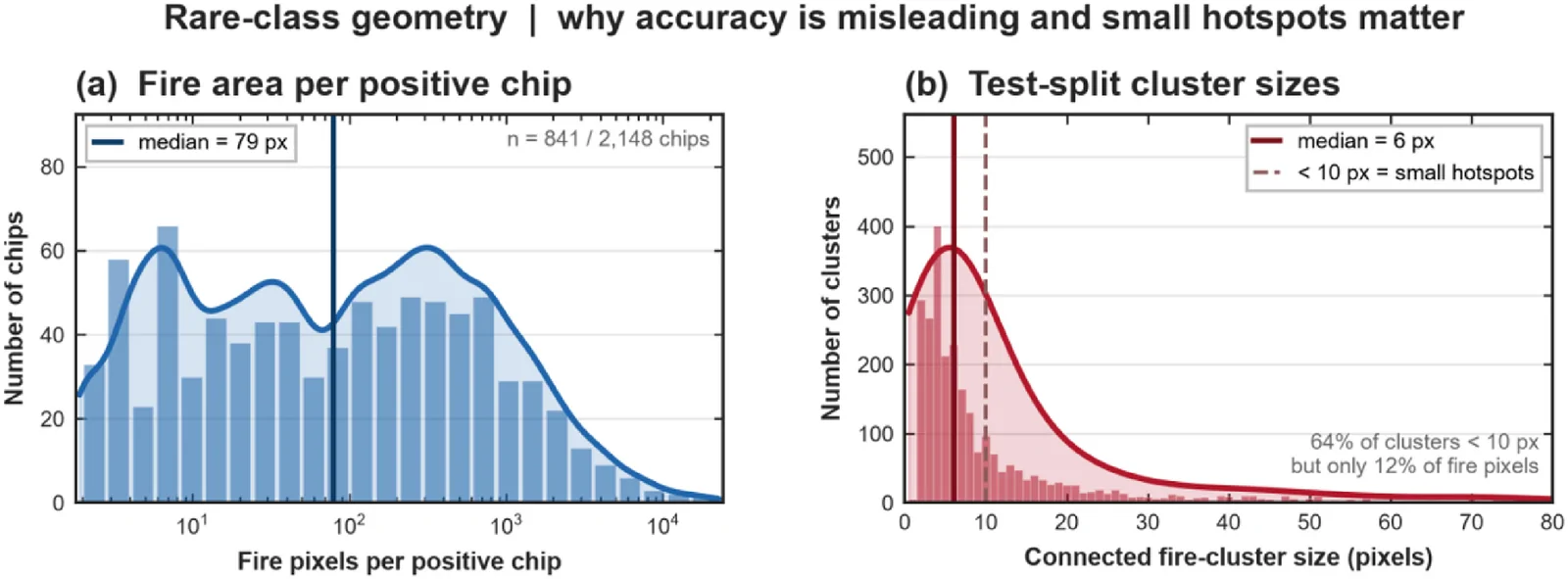
Spatial structure of the positive class. (a) Distribution of fire-pixel counts across the 841 fire-bearing chips; the horizontal axis is logarithmic and the vertical marker denotes the median of 79 pixels. (b) Distribution of connected fire-component sizes in the test partition, displayed up to 80 pixels. The solid and dashed markers indicate the six-pixel median and the ten-pixel size reference. The inset percentages refer to all test components and fire-labeled pixels, not only the displayed size range.

The median test component contains six pixels. Components smaller than ten pixels account for approximately 64% of test components but 12% of test fire-labeled pixels. Component counts and pixel counts describe different aspects of the targets. Comparisons at the component level should state the connectivity and matching rules.

At 20 m sampling, one grid cell represents a nominal 400 m² ground support. That support must not be interpreted as the physical area occupied by flame within the cell. Counts summed across chips and acquisition dates are pixel-observations: the same ground location can contribute more than once. They are therefore neither unique burned-area totals nor estimates of combustion area.

### 3.5 Reference training and label-review outputs

The companion implementation trains a segmentation model from the PNG pairs [2]. Fig. 6, Fig. 7, Fig. 8 show the reported reference run and the analyst-review results. The run documents one use of the dataset with the published partition and evaluation procedure. It does not compare architectures or measure operational fire-detection performance.

**Fig. 6:**
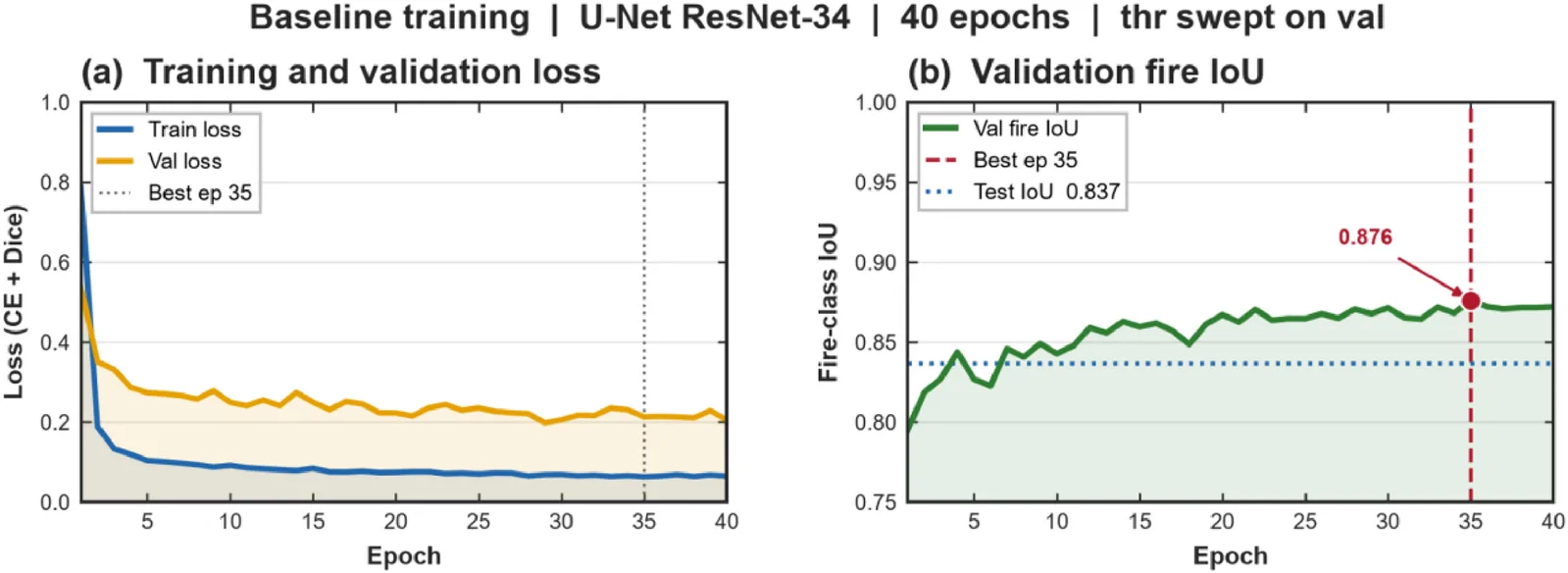
Reported 40-epoch reference run. (a) Training and validation losses for the weighted cross-entropy plus soft-Dice objective. (b) Validation fire-class intersection over union (IoU), using the threshold selected on validation at each epoch. The selected checkpoint is epoch 35, with validation IoU 0.876. The horizontal test reference, IoU 0.837, is shown for context and is not a checkpoint-selection criterion.

**Fig. 7:**
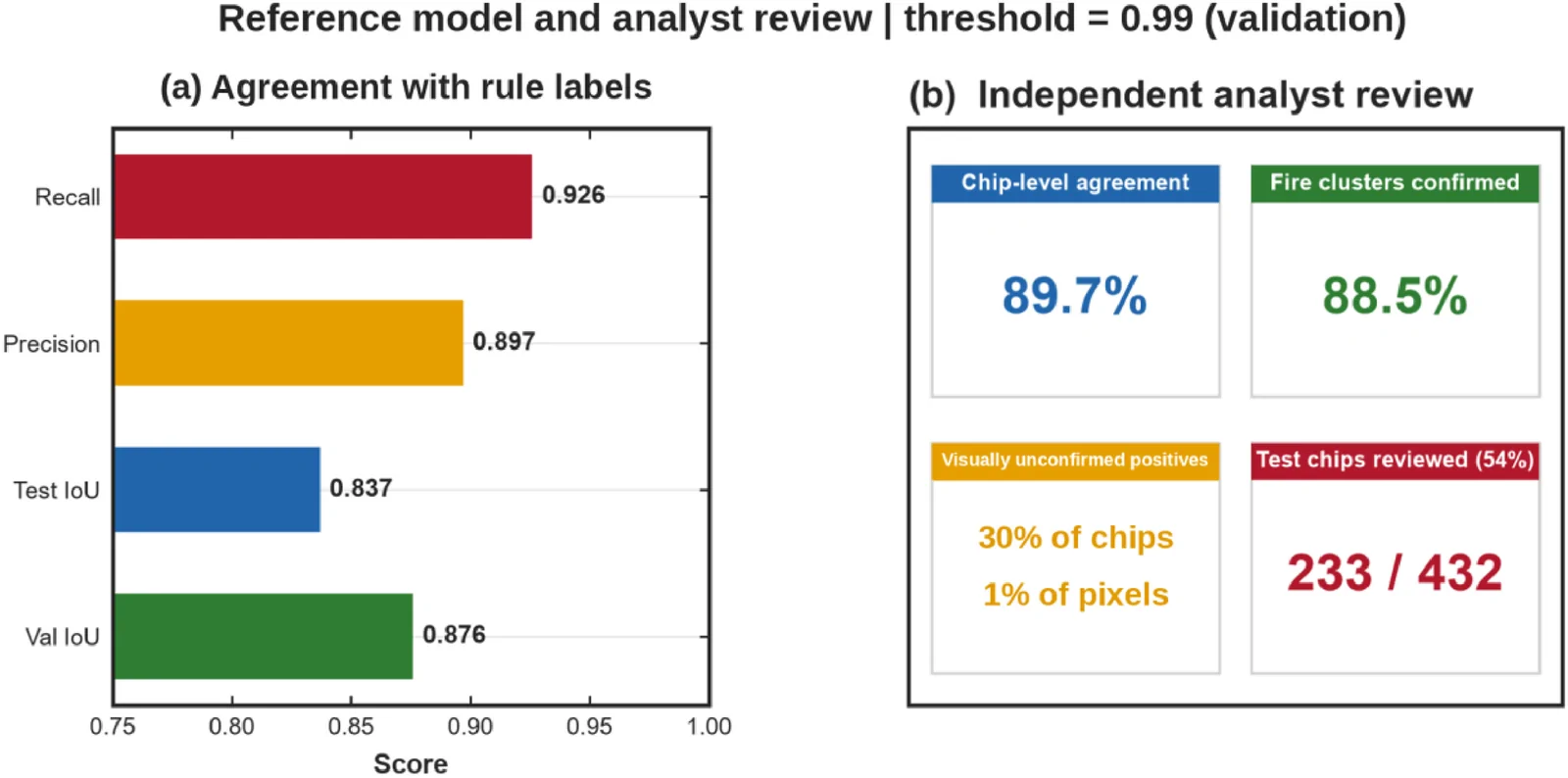
Reference-model agreement and analyst-review summary. (a) Validation IoU and test IoU, precision and recall against the SWIR-rule masks; the score axis begins at 0.75. (b) Review of 233 of 432 test chips. Chip agreement is 89.7%; 88.5% of rule components overlap an analyst fire annotation. Approximately 30% of reviewed rule-positive chips were visually unconfirmed; those chips contain approximately 1% of the rule-positive pixels in the reviewed batch. These two percentages have different denominators and do not describe a before-after reduction.

**Fig. 8:**
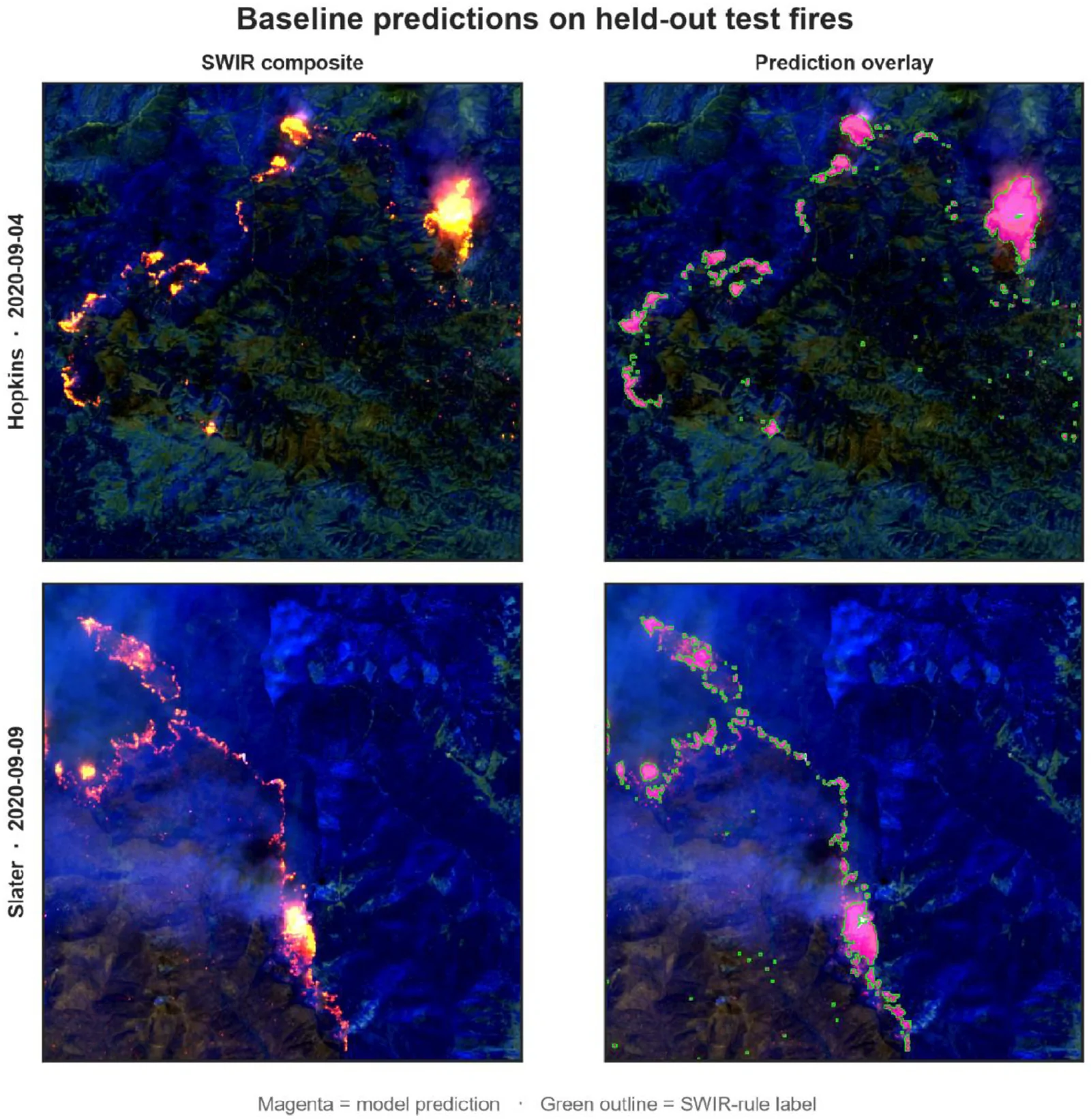
Reference predictions for Hopkins (4 September 2020) and Slater (9 September 2020). Left: SWIR composite. Right: the same view with model predictions shown in magenta and the SWIR-rule boundary in green. The green outlines identify the algorithmic reference, not an independent field or analyst delineation. Colors are visualization overlays; training uses the original composites and integer masks.

At the selected checkpoint, the validation-derived probability threshold is 0.99. Applying that operating point to the four test incidents gives a pooled fire-class IoU of 0.837, precision of 0.897 and recall of 0.926 against the distributed SWIR-rule masks (Fig. 7a). The score refers to the positive class and excludes invalid observations.

The analyst review is separate from the model evaluation. It covers 233 test chips (53.9%) and reports agreement with the algorithmic labels at chip and component levels (Fig. 7b). The subsection on analyst-review protocol and agreement measures describes sampling, uncertainty handling and component matching. Fig. 8 compares the model predictions and rule masks for two test wildfires.

### 3.6 Incident contributions

Fig. 9 lists active-fire pixel-observations by wildfire and partition. Counts vary with wildfire extent, sampled dates, cloud or smoke conditions and the position of active fronts within the sampling window. This distribution shows how much each wildfire contributes to a pooled pixel score. Evaluations that give equal weight to each wildfire can use the same inventory.

**Fig. 9:**
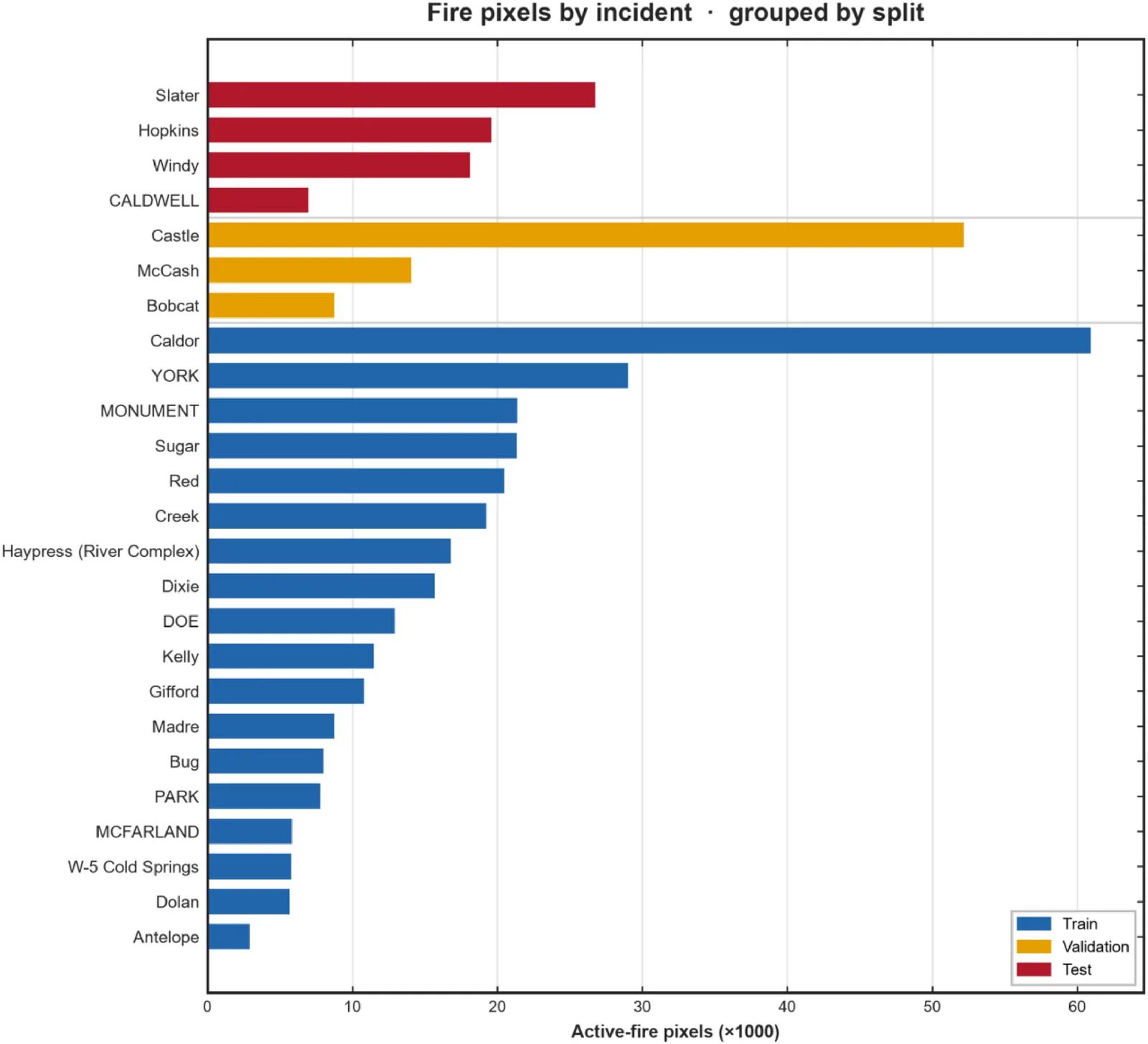
Contributions of active-fire pixel-observations by incident, grouped into test, validation and training partitions and ordered within each group. The axis is expressed in thousands of labeled pixels. Counts are accumulated over the sampled chips and dates; they do not represent unique burned area or a ranking of total incident size.

## 4 Experimental Design, Materials and Methods

### 4.1 Incident selection and acquisition sampling

Wildfire records were obtained from the NIFC WFIGS Interagency Perimeters service [8]. The query selected California records (attr_POOState = US-CA) with attr_IncidentTypeCategory = WF, discovery after 1 January 2019 and polygon area greater than 20,000 acres (approximately 80.94 km²). Prescribed fires, coded RX, were excluded. For repeated wildfire names, only the largest returned polygon was kept. We retained the 25 largest remaining wildfires by polygon area and verified all 25 names as WF on the WFIGS service. The records supplied wildfire names, discovery and containment dates, acreage and perimeters. This selection is not a census of all California wildfires.

For each wildfire, the sampling point was the midpoint of its geographic bounding box. The search window began at the recorded discovery date and ended at containment or 60 days after discovery, whichever came first. If containment was unavailable, it ended 45 days after discovery. A minimum window of seven days was enforced. Table 3 lists the acquisition and preparation settings.

**Table 3:** Acquisition and preparation settings in the public implementation.

Step	Setting or convention
Wildfire source	WFIGS Interagency Perimeters; California wildfire records only (attr_POOState = US-CA; attr_IncidentTypeCategory = WF; prescribed-fire RX excluded); post-1 January 2019 discovery; area >80.94 km²; 25 retained names.
Date window	Discovery to containment, capped at 60 days; 45-day fallback and seven-day minimum.
Scene source	Earth Search sentinel-2-l2a; point-intersection search; scene cloud cover ≤95%.
Scene selection	Lowest-cloud scene per date; at most 14 dates per incident.
Sampling grid	1536 × 1536 cells at 20 m, centered on the incident bounding-box midpoint and snapped in the selected scene’s projected coordinate system.
Chip geometry	Non-overlapping 3 × 3 subdivision into 512 × 512-cell chips within each parent window.
Footprint screen	Discard a chip if >50% of its cells are invalid.
Negative-chip selection	Retain all positive chips; apply integer-stride subsampling to the negative-chip list. Final archive: 841 positive and 1307 negative chips.

Sentinel-2 Level-2A scenes were queried through the public Earth Search STAC API (https://earth-search.aws.element84.com/v1, collection sentinel-2-l2a) [9]. Scenes had to intersect the wildfire sampling point and have reported cloud cover of 95% or less. For each date, only the scene with the lowest reported cloud cover was kept. Up to 14 dates were selected at approximately even index intervals from the chronological list. This samples the available dates across the window rather than at fixed calendar intervals. The scene-classification layer was not used to remove cloud or smoke pixels from the images or labels.

### 4.2 Radiometric conversion and image rendering

Bands B8A, B11 and B12 were read on the selected scene’s 20 m grid. Each band used the scale and offset in its STAC asset metadata; the fallback values were 0.0001 and zero when those fields were absent. Source digital number zero was treated as no data and reset to zero after conversion. The image and mask for a chip share the same grid. Each scene retains its projected coordinate system. Images from different dates are not stored as a coregistered geospatial stack.

The radiometric conversion and fixed rendering can be written as

(1) $$\;\rho_{b}=s_{b}D_{b}+o_{b},I_{b}=\text{round}\left[255\;\text{clip}\left(\rho_{b}/u_{b},0,1\right)\right]$$

In Eq. (1), $D_{b}$ is the source digital number in band b, $s_{b}$ and $o_{b}$ are that asset’s scale and offset, and $\rho_{b}$ is the resulting dimensionless Level-2A value for nonzero source digital numbers. The function clip(z,0,1) restricts z to the interval from zero to one; $I_{b}$ is the stored 8-bit channel value. The upper limits $u_{b}$ are 0.60 for B12, 0.50 for B11 and 0.45 for B8A. Channel order is B12-B11-B8A. These limits are display transformations, not the thresholds used to generate the labels. For a hot target, the converted optical-band signal can include emitted radiance and should not be interpreted as passive-surface reflectance alone.

### 4.3 Algorithmic label generation

The detector is a dataset-specific SWIR hot-target rule motivated by HOTMAP [7], not an unchanged implementation of the published algorithm. It operates on the converted band arrays before their clipping and quantization into PNG channels. Let the SWIR-to-NIR contrast be

(2) $$q=\left\{ \begin{array}{cc} \rho_{12}/\rho_{8A}, & \rho_{8A}>10^{-6},\\ +\infty , & \rho_{8A}\leq 10^{-6} \end{array}\right.$$

Here $\rho_{12}$ and $\rho_{8A}$ denote the converted B12 and B8A values at a pixel. The small denominator cutoff in Eq. (2) specifies the implementation’s numerical convention; it is not an independently validated physical boundary.

The seed set C and the more permissive candidate set P are

(3) $$C=\left\{\rho_{12}\geq 0.35\;\text{and}\;q\geq 4.0\right\},\;P=\left\{\rho_{12}\geq 0.25\;\text{and}\;q\geq 2.0\right\}$$

Thus, C contains high-contrast seed pixels and P defines where those seeds may expand. All thresholds refer to the converted values, not to the 0–255 rendered channels. B11 supplies image context and participates in the validity test, but no B12/B11 condition is used in Eq. (3).

Growth is limited to two four-neighbor steps:

(4) $$G_{0}=C,\;\;G_{k+1}=G_{k}\cup \left[\delta_{4}\left(G_{k}\right)\cap P\right],\;\;k=0,1$$

In Eq. (4), $G_{k}$ is the grown set after k steps and $\delta_{4}$ is a one-pixel dilation using horizontal and vertical neighbors. The intersection with P constrains each step to candidate pixels. An eight-connected binary sieve with a two-pixel size parameter is then applied to $G_{2}$ using rasterio.features.sieve [2]. Growth stops after two steps; it does not continue through the entire connected region.

A cell is treated as valid when each of the three converted band values is nonzero, following the released implementation. The final mask assigns 1 to detected fire in valid cells, 0 to remaining valid cells and 255 to cells failing that validity test. The code 255 marks invalid cells, mainly where acquisition coverage is missing. It does not identify clouds. A valid cell labeled 0 means that the rule did not identify active fire there. It does not confirm the absence of combustion.

### 4.4 Tiling, sampling and evaluation partitions

For each scene, a 1536 × 1536-cell window at 20 m was centered on the wildfire’s bounding-box midpoint and snapped to the scene’s pixel grid in its projected coordinate system. This 30.72 km-wide window was divided into a non-overlapping 3 × 3 grid of 512 × 512-cell chips, each 10.24 km wide. A chip was discarded if >50% of its cells failed the validity test described under algorithmic label generation. All fire-bearing chips were kept. The fire-free chip list was subsampled using an integer stride calculated from the positive and negative counts [2]. The configured negative-to-positive ratio was one, but integer-stride selection does not produce exact equality. The archive contains 841 fire-bearing and 1307 fire-free chips (Table 1).

Partition membership is assigned by wildfire name using the validation and test names listed under release organization and partition inventory. All chips from the same wildfire stay in one partition. Image normalization is estimated from training data, and augmentation uses training chips only. Grouped evaluation is appropriate when observations within a group share spatial or temporal structure [10]. Splitting by wildfire name prevents the same wildfire from appearing in training and evaluation sets. It does not impose a geographic buffer between different wildfires.

Use the archived manifest for comparisons with this release. The source services can change, so rerunning the queries may produce a different scene selection and different files. To reconstruct geospatial rasters, retain the source scene identifiers, coordinate reference systems and grid transforms. These are not embedded in the PNG files or the six-field chip manifest.

### 4.5 Analyst-review protocol and agreement measures

The review batch was drawn from the four test incidents and included both rule-positive and rule-negative chips. Tasks were presented in a fire-stratified randomized order. The analyst inspected SWIR composites without the generated masks or their fire-pixel counts. Training-chip examples were used to explain the annotation convention. The protocol asked the analyst to identify visible active-fire cores, leave nonfire regions unmarked and use an uncertainty label where a reliable judgment could not be made [2].

The initial labeling canvas used a twofold nearest-neighbor enlargement. Exported brush masks were mapped back to 512 × 512 cells. A native cell was assigned to fire when any corresponding higher-resolution subcell was marked as fire; uncertainty took precedence where the classes overlapped. The importer also used the annotation export to retain reviewed empty chips. For comparisons, cells marked 255 by either the rule mask or the analyst mask were excluded.

Chip agreement compares the presence or absence of at least one fire cell in the two masks over the retained cells. A rule component is confirmed when any of its cells overlaps an analyst fire annotation. Components use eight-neighbor connectivity, with no additional spatial tolerance in the matching step [2]. The reported component-confirmation fraction is

(5) $$A_{\text{component}}=\frac{1}{K}\sum_{j=1}^{K}1\left(C_{j}\cap H\neq \emptyset \right)$$

In Eq. (5), K is the number of rule components across the reviewed, comparable cells, *C* is the set of cells in component *j, H* is the analyst fire-cell set in the corresponding chip, and **1** is an indicator equal to one when the condition is true and zero otherwise. This criterion evaluates overlap with an analyst delineation; it does not require one-to-one correspondence between components and can depend on delineation width.

The 233-chip review yielded 89.7% chip agreement and 88.5% rule-component confirmation. The approximately 30% visually unconfirmed fraction uses reviewed rule-positive chips as its denominator. The approximately 1% pixel fraction uses all rule-positive pixels in the reviewed batch as its denominator and counts those lying in visually unconfirmed chips. It is not an estimate that only 1% of all labeled pixels are incorrect. These results apply to the reviewed subset. They are not field-confirmed detection probabilities for all test observations.

### 4.6 Reference segmentation configuration

A U-Net [11] with a ResNet-34 encoder [12] is the reference model. The implementation supports ImageNet initialization [13] and two output classes. Invalid cells are excluded from optimization. Table 4 lists the reference settings. The model provides an example of training with the released data.

**Table 4:** Reference training and evaluation settings. These settings describe the public script and the operating point reported in Fig. 6, Fig. 7, Fig. 8. New runs should record the backend, software versions and saved checkpoint.

Component	Setting
Input and output	Three rendered channels, normalized with training-derived statistics; two class logits.
Architecture	ResNet-34 U-Net implemented with the torchvision fallback used for the reported run (segmentation_models_pytorch is preferred by the script when installed, but was not the backend for the archived checkpoint). ImageNet initialization via torchvision IMAGENET1K_V1.
Optimization	AdamW [14]; initial learning rate 0.0003; weight decay 0.0001; cosine learning-rate annealing.
Training schedule	40 epochs; batch size 8; encoder frozen for two epochs, then unfrozen at 0.1 times the initial base learning rate.
Sampling	Replacement sampling; weight 3 for positive chips and 1 for negative chips; four draws per training chip per epoch on average.
Geometric augmentation	Flips and 90-degree rotations; crop scale 0.55–1.35; fire-centered crops with probability 0.85 when a positive cell is available.
Copy-paste	Probability 0.5; one to three donor chips; two-pixel feathered context halo; donors restricted to training chips.
Photometric augmentation	Common-channel gain within ±12%; additive noise with standard deviation 0.02 on the 0–1 image scale.
Loss	Class-weighted cross-entropy plus fire-class soft Dice, with Dice weight 1 and smoothing constant 1.
Numerical settings	Mixed precision on CUDA; gradient-norm clipping at 5; configured seed 0. Reported run: Kaggle NVIDIA Tesla T4; exact container environment documented in docs/REFERENCE_RUN.md.
Selection	Maximum pooled validation fire IoU over checkpoints and a fixed threshold grid.
Reported operating point	Epoch 35; probability threshold 0.99; validation fire IoU 0.876.

Training samples are augmented on the fly; the distributed images and masks are not overwritten. Copy-paste follows the general use of composited labeled instances for augmentation [15], adapted here to fire regions with a surrounding blending halo. Donors are drawn only from positive training chips. Mask handling preserves categorical values; when zooming out, the code uses coverage-based retention to avoid dropping sparse positive cells [2].

The optimization objective combines weighted cross-entropy with a soft-Dice overlap term, following the use of overlap-based losses for segmentation [16]:

(6) $$L=-\frac{1}{\left|V\right|}\sum_{i\in V}w_{y_{i}}\text{log}p_{i,y_{i}}+1-\frac{2\sum_{i\in V}p_{i,1}y_{i}+1}{\sum_{i\in V}+\sum_{i\in V}y_{i}+1}$$

Here V is the set of valid cells in the batch; |V| is its size; $y_{i}\in \left\{0,1\right\}$ is the rule label; and $p_{i,c}$ is the softmax probability for class c at cell i. The cross-entropy weight is $w_{0}=1$ for background and $w_{1}=\text{min}\left(50,\text{max}(1,\sqrt{1/f_{s}})\right)$ for fire, where $f_{s}$ is the expected fire-cell fraction under the training sampler before augmentation. The constants of one in the overlap ratio are the implementation’s smoothing terms. Equation (6) specifies the released linear-sum Dice denominator explicitly.

### 4.7 Checkpoint selection and reference evaluation

At each epoch, validation probabilities are evaluated at thresholds 0.05, 0.10, …, 0.90, together with 0.95, 0.97, 0.98, 0.99 and 0.995. The checkpoint and threshold giving the highest pooled validation fire-class IoU are retained. The reported test scores use that validation-selected operating point, without selecting a threshold to maximize the reported test result.

For all valid cells pooled across the evaluated partition,

(7) $$\text{IoU}=\frac{TP}{TP+FP+FN},\;\text{Precision}=\frac{TP}{TP+FP},\;\text{Recall}=\frac{TP}{TP+FN}$$

In Eq. (7), TP counts cells positive in both the model prediction and the rule mask, FP counts predicted fire in rule-background cells, and FN counts rule-fire cells predicted as background. A model cell is positive when its fire probability is at least the selected threshold. Counts are accumulated across the partition before division; the reported IoU is not a mean of per-chip IoUs. These quantities measure model agreement with the algorithmic reference and are distinct from the analyst-review quantities in Eq. (5).

## Limitations

The active-fire labels are derived from the same optical bands used in the images. Model agreement with these labels measures reproduction of the SWIR rule. The analyst review provides a separate visual assessment of 233 test chips. It does not establish field-confirmed pixel boundaries or agreement between multiple analysts.

The dataset samples large California wildfires. The fixed parent windows may cover only part of a wildfire perimeter, and splitting by wildfire name does not ensure a geographic gap between neighboring fires. Cloud and smoke can obscure active fronts. The selected acquisitions do not capture continuous or diurnal fire evolution. The 8-bit images are intended for the documented segmentation task; quantitative spectral analysis requires the original radiometric assets. PNGs also omit geospatial transforms. Geographic transfer or operational fire-detection studies require separate validation for their intended use.

## Ethics Statement

The authors have read and follow the ethical requirements for publication in Data in Brief. This work does not involve human subjects, animal experiments, or data collected from social media platforms. It uses satellite imagery and publicly accessible incident-perimeter records; analyst annotations are data-curation outputs.

## CRediT Author Statement

**Shreyan Mitra:** Conceptualization, Methodology, Software, Validation, Formal analysis, Investigation, Data curation, Writing - original draft, Visualization. **Mohammadreza Narimani:** Writing - review & editing, Visualization, Project administration. **Parastoo Farajpoor:** Writing - review & editing, Supervision.

## Generative AI statement

During the preparation of this work, the authors used ChatGPT to assist with language editing and refinement of visualization code. The authors reviewed and edited all resulting content and take full responsibility for the content of the article.
